# Dietary inclusion of *Morus alba* leaves influences milk production and offspring growth performance in dairy goats

**DOI:** 10.1007/s11250-026-05109-3

**Published:** 2026-06-15

**Authors:** Hanan A. M. Hassanien, Nehal M. El-Hendawy, Liza A. Abdel-Rafaa, Mohamed M. El‑Badawy, Hanim A. ElSheikh, Ezzat Arafa El-Bltagy, Sherif Abdelghany, Mohamed A. Radwan, Moisés Cipriano Salazar, José Luis Ponce-Covarrubias, Abdelfattah Z. M. Salem

**Affiliations:** 1 Animal Production Research Institute, Agricultural Research Center, Giza, 12619 Egypt; 2 Department of Animal Production, Faculty of Agriculture, Cairo University, Giza, Egypt; 3 Facultad de Medicina Veterinaria y Zootecnia, Carretera Cd. Altam.-Iguala, Km3, Colonia Querenditas, Cd. Altamirano, Guerrero, México; 4 Escuela Superior de Medicina Veterinaria y Zootecnia No. 3, Universidad Autónoma de Guerrero (UAGro), Técpan de Galeana, Guerrero, México; 5 Dipartimento di Scienze del Suolo, della Pianta e degli Alimenti (Di.S.S.P.A.), Università degli Studi di Bari, Via Giovanni Amendola, 165/a, 70126 Bari, BA Italy

**Keywords:** Goats, Mulberry leaves, Economic efficiency, Feed efficiency, Milk yield, Milk quality, Reproductive

## Abstract

This study evaluated the effects of incorporating *Morus alba* (mulberry, “ML”) leaves into the diets of Damascus does and their offspring on productive, reproductive, and economic performance. Thirty-six multiparous Damascus does in the last third of pregnancy were randomly allocated to three dietary treatments for 150 days: a control group (ML0), a group in which 25% of the diet was replaced with mulberry leaves (ML25), and a group in which 50% of the diet was replaced with mulberry leaves (ML50). The feeding trial extended throughout late gestation and the suckling period, whereas reproductive performance was evaluated during the subsequent postpartum breeding season. Results indicated that ML inclusion significantly enhanced nutrient digestibility and optimized rumen fermentation, as evidenced by increased volatile fatty acids and lower ruminal ammonia levels. Milk yield and 4% fat-corrected milk showed only numerical, non-significant increases, while fat yield was improved despite lower milk fat and protein percentages in the mulberry-supplemented groups. Reproductive performance also improved, with the ML50 group showing enhanced estrus response, elevated progesterone levels, and better fertility rated outcoms during the subsequent postpartum breeding season. Kids from the supplemented groups showed significantly higher weaning weight, whereas total weight gain and average daily gain increased numerically but did not reach statistical significance. Economically, the ML50 treatment yielded the highest efficiency and strongest financial outcomes. In conclusion, replacing 50% of the diet with mulberry leaves may offer a sustainable and cost-effective strategy to improve goat productivity by supporting rumen function, fat yield, offspring weaning performance, postpartum reproductive responses, and economic returns.

## Introduction

In several countries, livestock farming, especially sheep, goats and deer, is an important part of life, where a prominent portion of agricultural societies depends on them and their derived products (Mazinani and Rude 2020; Rodríguez Huerta et al. 2026). Conversely, ruminant producers face considerable challenges directly due to a lack of feed and rising costs, especially in the dry season (Duguma and Janssens 2021; D’Alessandro et al. 2026; Rodríguez Huerta et al. 2026). The ability of livestock to respond positively to various feed additives can significantly improve their overall performance by reducing metabolic risks and enhancing nutrient use efficiency (Nian et al. 2017; Botia Carreño et al. 2026). Incorporation of tree leaves and crops by-products can positively impact small ruminants’ sustainability (Farghaly et al. 2022; Adegbeye et al. 2025), enabling farmers to create cost-effective rations, recycle by-products, and produce safe food (Boudalia et al. 2024; Hassanien et al. 2026b).

Mulberry (shrub or tree) has been historically utilized in sericulture across several countries. It is classified under the order Urticales, the Moraceae (Family), and the Morus (genus). The most common species include *M. alba* (white mulberry), *M. rubra* (red mulberry), *M. nigra* (black mulberry), and M. indica. These plants serve as valuable resources, often used as feed for farm animals, boosting animal productivity, feed efficiency, and meat quality (Cai et al. 2019; Liu et al. 2019).

The leaves of the mulberry tree are a significant source of natural food additives, known for generating lush green foliage, and are rich in numerous nutrients (Xue et al. 2025). These leaves are rich in protein content, which is partly soluble in the rumen, and in insoluble carbohydrates (Tan et al. 2012). Besides, they have significant levels of sulfur and other principal minerals, are free of harmful substances, and are highly palatable to ruminant animals (Li et al. 2020). Furthermore, mulberry leaves enhance nutrient digestion and rumen microbial activity by optimizing the rumen environment (Huyen et al. 2012).

Mulberry ranks among plant-based products as one of the highest nutritional values, considerably superior to that of traditional forages and potentially the quality of concentrates. Also, Mulberry leaves (ML) are highly digestible, with an impressive crude protein (CP) content ranging from 23% to38% (Yan et al. 2024). Guo et al. (2025) evaluated the mulberry nutritional profile and found that the composition of its leaves and cell wall constituents, along with carbohydrates and ash contents, confirms that mulberry is an incomparable feed for high-performing livestock. It is proper for both fresh and dried purposes for feeds. ML performs a key role in encouraging growth and improving milk production as a result of its nutritional contents (Chandra et al. 2023). ML as a feed supplement for dairy cows has been widely adopted due to their competence to increase milk production with feed cost reduction (Hassan et al. 2020).

Flavonoids are an important group of natural, plant-derived polyphenolic combinations, commonly found in animal feed and forage (Botia-Carreño et al. 2025; Elghandour et al. 2026). Consequently, animals consume them in various amounts as a component of their usual diet; nevertheless, the concentrations in feed plants are relatively modest. Flavonoids are widely documented as effective feed supplements, related to their notable biological properties, which enhance both livestock performance and the quality of animal-derived products (Archundia Velarde et al. 2025; Hassanien et al. 2025, 2026a). Flavonoids derived from plant species within the genus Morus are particularly acclaimed for their antioxidant properties. Combining ML in a livestock feeding system has been exhibited to advance overall performance by increasing feed intake, improving digestion and nutrient absorption, supporting mammary gland maturation, and promoting immune resilience (Geng et al. 2024). The justification for this study arises from the urgent need to address the rising costs of conventional feed and the scarcity of resources, which challenge the sustainability of livestock farming (Duguma and Janssens 2021). Mulberry leaves (Morus alba) present a promising alternative due to their exceptional nutritional profile and rich concentration of bioactive compounds (Hassan et al. 2020; Yan et al. 2024). We hypothesize that replacing a significant portion of concentrate feed with mulberry leaves will optimize rumen fermentation kinetics and enhance antioxidant status. Consequently, this is expected to improve milk quality, reproductive efficiency, and the growth performance of offspring, providing a sustainable nutritional strategy. Therefore, the current study aimed to evaluate the impact of incorporating mulberry leaves into the diets of Damascus goats on their productive and reproductive performance, as well as the growth performance of their suckling kids.

## Materials and methods

### Ethical approval and experimental site

All animal procedures were approved by the Cairo University Institutional Animal Care and Use Committee (CU IACUC; approval reference: CU-II-F-43-24). Current research was conducted at the El-Gemaisa Animal Production Research Station, which is affiliated with the Animal Production Research Institute (APRI), Ministry of Agriculture, Egypt.

### Mulberry leaves

Fresh leaves were collected from ten mature mulberry trees (5–7 years old) at the experimental farm of the Animal Production Research Institute (APRI) in El-Gemaysa, Egypt, during the vegetative period (June–July). Only healthy, disease-free leaves were harvested manually, primarily from the middle third of the branches. Immature apical and old basal leaves were excluded to ensure equal nutritional composition. A sufficient number of leaves was collected to make sure that the experimental diets were all the same, air-dried in the shade at ambient temperature (25–28 °C) with adequate ventilation to preserve nutrients and pigments, and subsequently crushed to pass through a 1-mm screen. The desiccated substance was stored until it was required for the preparation of the experimental rations.

### Active compounds in mulberry leaves

High-Performance Liquid Chromatography parameters. An Agilent 1260 series apparatus was used for HPLC analysis. The separation was achieved on a Zorbax Eclipse Plus C8 column (4.6 × 250 mm, 5 μm, particle size). The mobile phase comprised water (A) and 0.05% trifluoroacetic acid in acetonitrile (B) at a flow rate of 0.9 ml/min. The mobile phase was sequentially programmed in a linear gradient as detailed below: 0 min (82% A); 0–1 min (82% A); 1–11 min (75% A); 11–18 min (60% A); 18–22 min (82% A); 22–24 min (82% A). The multi-wavelength detector was observed at 280 nm. The injection volume for each sample solution was 5 µl. The column was kept at a constant temperature and sustained at 40 °C (Ramadan et al. 2024).

### Animal life, environmental conditions, and research methodology

This study involved thirty-six clinically healthy, multiparous Damascus dairy goats in the last third of gestation,aged 4 to 7 years and in their second to fifth parities, with an average live body weight of 42.13 ± 0.27 kg. Based on parity and initial body weight, the animals were randomly allocated into three experimental groups, each comprising 12 does. The nutritional intervention started during late gestation and continued after kidding throughout the 90-day suckling period. Productive performance was evaluated through dam body weight, milk production, milk composition, and offspring growth, whereas reproductive performance was assessed after kidding during the subsequent postpartum breeding season. During the trial, all the does were housed in semi-open, shaded barns under uniform environmental and management conditions. The housing provided adequate floor space per animal, with natural ventilation and lighting. Animals were grouped accoding to their assigned dietary treatments; however, feeding was conducted individually for each animal to ensure accurate control of feed intake. All animals were provided with clean bedding, continuous access to potable water, and regular health monitoring, and were maintained free of internal and external parasites throughout the trial. The dam and her associated litter constituted the experimental unit for assessing productive performance, reproductive traits, and offspring growth. This approach allowed evaluation of the effect of maternal nutrition on dam productivity and offspring performance under practical production conditions.

### Diets and feeding management

The feeding trial started during the last 60 days of gestation and continued after kidding throughout the 90-day suckling period until weaning. Milk production and offspring growth were monitored during the suckling period, whereas postpartum reproductive performance was evaluated during the subsequent breeding season. Nutrient requirements were modified biweekly based on fluctuations in body weight, reproductive phase, and milk yield. The goats’ principal diet was a concentrate feed mixture that accounted for 60% of their intake. Each goat was administered 250 g of rice straw daily at 08:00 h. Fresh berseem (*Trifolium alexandrinum*) was offered ad libitum on an individual basis. Animals were randomly allocated into three groups based on their diets: the control group (ML0), which received the standard ration devoid of mulberry leaves; the ML25 group, which had 25% of the concentrate feed mixture replaced with mulberry leaves; and the ML50 group, which had 50% of the concentrate feed mixture replaced with mulberry leaves. Table 1 delineates the chemical makeup of feed ingredients, concentrate combinations, and experimental rations computed on a dry matter basis.

**Table 1:** Chemical composition of feedstuffs, concentrate feed mixtures, and calculated chemical composition1 of experimental rations (% on DM basis)

Item^1^	CFM^2^	RS	Berseem^3^	Mulberry leaves	CFM 25% Mulb^4^	CFM 50% Mulb	ML0^5^	ML2^6^	ML50^7^
DM	91.8	90.6	14.5	88.8	91.1	90.0	76.5	76.0	75.4
OM	88.2	84.7	88.4	79.3	85.9	83.7	87.6	86.2	84.8
CP	14.1	3.4	16.4	16.2	14.6	15.1	12.8	13.2	13.9
CF	8.9	37.0	25.9	16.0	10.6	12.4	15.1	16.3	17.4
EE	2.8	3.1	1.5	3.1	2.9	3.0	2.3	2.3	2.4
NFE	62.4	44.0	44.7	44.0	57.8	53.2	57.4	54.4	51.5
NDF	26.9	72.8	40.7	36.7	29.4	31.8	38.8	40.3	41.8
ADF	26.5	49.9	28.6	23.7	25.8	25.1	31.7	31.1	30.8
ADL	4.4	10.6	5.6	6.1	4.8	5.5	5.9	6.1	6.3

^1^DM: Dry matter; OM: organic matter; CP: crude protein; CF: crude fiber; EE: ether extract; NFE: nitrogen-free extract; NDF: Neutral detergent fiber; ADF: Acid detergent fiber; ADL: Acid detergent lignin

^2^CFM: Concentrate feed mixture; ^3^Berseem: (*Trifolium alexandrinum*), ^4^Mulb: mulberry leaves; ^5^ML0: basal ration (60% concentrate feed mixture (CFM) plus 250 g rice straw/goat/day alongside Berseem as *ad libitum*; ^6^ML25; basal ration (ML0) with 25% of the CFM substituted by mulberry leaves; ^7^ML50: basal ration (ML0) with 50% of the CFM replaced by mulberry leaves

### Nutrient digestibility trial and ruminal fermentation

A distinct digestibility trial was performed on adult Damascus bucks to assess food digestibility and ruminal fermentation characteristics, while excluding the influences of physiological alterations related to pregnancy and lactation. Nine clinically healthy bucks, roughly 24 months old and weighing 50 kg each, were employed in the study. Participants were randomly assigned to one of three eating regimens, with three bucks provided per treatment, yielding three replicates for each ration. Bucks were separately housed in metabolic enclosures that enabled the assessment of their food consumption and fecal production. The pens demonstrated enough airflow, upheld cleanliness, and ensured unobstructed access to drinking water. The digestibility experiment had a 14-day acclimatization phase succeeded by a 7-day collection period. Daily fecal samples were obtained from each animal. 10% of these samples were combined for each animal and preserved at − 20 °C until testing could be performed. Feed, refusals, and fecal samples were desiccated in an oven at 60 °C for 72 h, crushed with a 1-mm screen using a Wiley mill, and later analyzed for chemical composition according to AOAC (1990) criteria. We utilized the acid-insoluble ash method described by Van Keulen and Young (1977) to determine the nutrient digestibility coefficients. Subsequent to the digestibility evaluation, stomach tubes were utilized to obtain rumen liquor samples from all bucks at 0, 3, and 6 h after intake. The samples were filtered through three layers of cheesecloth, and the ruminal pH was promptly measured. The concentration of ammonia nitrogen (NH₃-N) was assessed using the method outlined by Conway (1962), and the total volatile fatty acids were measured in accordance with Warner (1964).

### Reproductive performance

Postpartum reproductive performance was evaluated during the subsequent mating season after kidding. Estrus detection lasted for 15 days, beginning at the end of August, and was followed by a 45-day mating season from early September to mid-October 2024. Estrus was detected by using a teaser on the bucks twice a day (06:00–08:00 and 15:00–16:00). The does’ body weight (BW) and estrus incidence data were recorded. Estrus-exhibiting does were mated by a buck twice daily (8:00 a.m. and 3:00 p.m.). Several postpartum reproductive traits, including estrus response, mating, conception, pregnancy, kidding, fecundity, prolificacy, and twinning rates, were recorded for each treatment during the subsequent breeding cycle.

### Milk yield and quality

Both the does and their kids were weighed at 15 h postpartum and then biweekly over 90 days until the kids were weaned. Milk yield was estimated by weighing the kids before and after suckling, following eight hours of separation from their dams (Abd-Allah et al. 2011). Milk samples were collected biweekly via hand milking for milk quality analysis. Fat, protein, lactose, and total solids percentages were determined using a Milko-Scan analyzer (Foss Electric, Hillerød, Denmark). Solids-not-fat (SNF) was calculated by subtracting milk fat percentage from total solids percentage according to the following equation: SNF (%) = total solids (%) − fat (%). The fat-corrected milk 4% (FCM) for goats was computed by the equation of Mavrogenis and Papachristoforou (1988): FCM = milk yield × (0.411 + 0.147 × fat percentage).

### Feed efficiency and economic analysis

Feed efficiency was calculated based on the quantity of dry matter (DM), total digestible nutrients (TDN), and digestible crude protein (DCP) units per kg of milk yield. Economic efficiency was calculated as the ratio between total revenue and total feeding costs during the experimental period in 2024. The input costs include the prices of the concentrate feed mixture, Egyptian clover, rice straw, and mulberry leaves, which were 14,000, 900, 400, and 1500 LE/ton, respectively. The income includes the LBW price (180 LE/kg).

### Progesterone (P4) analysis

During the subsequent postpartum mating season, blood samples were collected from the jugular vein of each doe at 10-, 35-, and 50-days post-mating. Following clot formation, all samples were centrifuged at 1,000 x g for 15 min. Serum was kept at -20 °C until the P4 analyses. A radioimmunoassay kit supplied by Immunotech, France (catalog No. IL88) to measure serum P4 concentrations. A 50 µL aliquot of each sample was mixed with 500 µL of labeled P4 as a tracer in an antibody-coated tube and incubated for 1 h. Serum P4 levels were measured using an automatic Mini-Gamma counter (plate number 1, VSA). Assay sensitivity was 0.03 ng/mL, with intra- and inter-assay coefficients of variation of 4.3%.

### Blood parameters analysis

Samples were drawn from the jugular veins of does at the end of the experimental period. To acquire blood serum, samples were centrifuged for 20 min at 3000 rpm, then the supernatant was stored at-20 °C for later analysis. The analysis was performed according to manufacturing guidelines using a kit (Diamond Diagnostics, Egypt), a colorimetric technique to measure total protein (TP) level, albumin concentrations, while serum globulin (GL) was calculated as subtraction between TP and albumin, alanine aminotransferase (ALT); Aspartate aminotransferase (AST), urea, creatinine level, triglyceride, total cholesterol level. Finally, total antioxidant capacity (TAC).

### Statistical analysis

The general linear model (GLM) procedure was used to analyze all the data (SAS 2000). Prior to analysis, percentage data were transformed using the arcsine function to improve normality. Significant treatment means were assessed via the Duncan test (Duncan 1955). The statistical model was: Y_ij_ = µ + T_i_ + e_ij_, where: Y_ij_ = the response variable, µ = the overall mean, T_i_ = the effect of the treatment i, and e_ij_ = the random error.

## Results

### Active compound in mulberry leaves

**Fig. 1:**
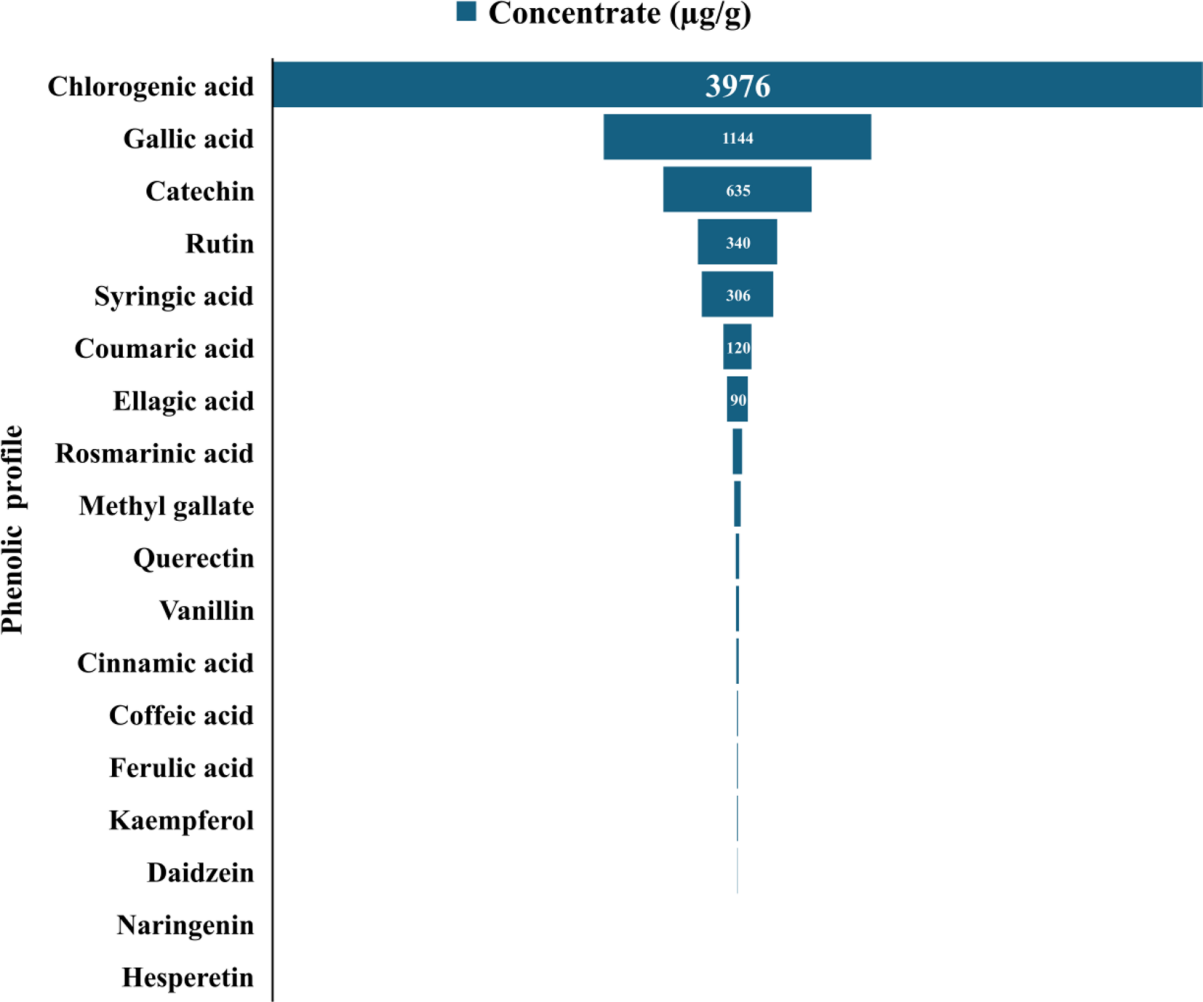
The phenolic content profile and concentration (µg/g) in *mulberry alba* leaves

Figure 1 illustrates the phenolic chemical profile of mulberry leaves. Types and quantities of secondary metabolites exhibit significant variation. Chlorogenic acid (3976 µg/g) was the predominant component, followed by gallic acid (1144 µg/g), syringic acid (764 µg/g), and catechin (635 µg/g). Alongside these main components, moderate quantities of rutin (340 µg/g), coumaric acid (120 µg/g), and ellagic acid (90 µg/g) were recognized. Trace quantities (< 50 µg/g) of rosmarinic acid, methyl gallate, quercetin, vanillin, cinnamic acid, caffeic acid, ferulic acid, kaempferol, and daidzein were also identified.

### Digestion coefficients and nutritive values

The data presented in Table 2 show that inclusion of ML in the diet resulted in more easily digestible organic matter (OM), crude protein (CP), and crude fiber (CF). This finding indicated that a dose-dependent association between the effect of ML inclusion levels and nutritional digestion and absorption. Conversely, no significant variation was observed in the digestibility coefficients of dry matter (DM), ether extract (EE), or nitrogen-free extract (NFE), which remained similar in all experimental groups. There were significant differences in TDN, DCP, and digestible crude protein intake (DCPI) compared to the control group (ML0). However, adding mulberry leaf did not have a significant impact on intakes of DM or TDN. The digestibility of neutral detergent fiber (NDF) and acid detergent fiber (ADF) significantly increased with the addition of additional ML, indicating an enhancement in the fiber component of the diet. The findings combined suggest that including ML into ruminant diets can improve nutrient digestibility, particularly for protein and fiber components, hence increasing the nutritional efficiency of the feed.

**Table 2:** Digestibility coefficients and feeding values of experimental rations^1^

	ML0	ML25	ML50	SEM	*P* value
Digestibility coefficients, %^2^					
DM	61.5	63.0	63.1	0.4	0.091
OM	65.3^c^	66.4^b^	68.1^a^	0.2	0.001
CP	63.8^c^	65.8^b^	68.2^a^	0.1	0.001
CF	56.7^c^	59.4^b^	62.4^a^	0.2	0.001
EE	71.7	74.5	76.2	1.8	0.301
NFE	67.6	68.3	69.7	2.7	0.687
NDF	53.3^c^	55.5^b^	58.4^a^	0.7	0.021
ADF	47.7^c^	49.8^b^	52.2^a^	0.9	0.013
Feeding values, %^2^					
DMI, g	1226	1211	1192	45.7	0.871
TDN, %	59.3^c^	59.4^b^	60.0^a^	0.3	0.001
DCP, %	8.2^b^	8.7^b^	9.2^a^	0.1	0.001
TDNI, g	726.4	719.1	714.9	37.5	0.875
DCPI, g	100.2^b^	104.7^b^	109.5^a^	6.3	0.045

^1^ML0: basal ration (60% concentrate feed mixture (CFM) plus 250 g rice straw/goat/day with Berseem (*Trifolium alexandrinum*) as *ad libitum*); ML25; basal ration (ML0) with 25% of the CFM substituted by mulberry leaves (ML); ML50: basal ration (ML0) with 50% of the CFM replaced by ML

^2^DM: dry matter, OM: organic matter, CP: crude protein, CF: crude fibre, EE: ether extract, NFE: nitrogen-free extract, NDF: Neutral detergent fiber; ADF: Acid detergent fiber, DMI: Dry matter intake, TDN: total digestible nutrients, DCP: digestible crude protein, TDNI: total digestible nutrient intake, and DCPI: digestible crude protein intake

a, b and c mean in the same row with different subscripts differed significantly (*P* < 0.05)

### Rumen fermentation activities

**Fig. 2:**
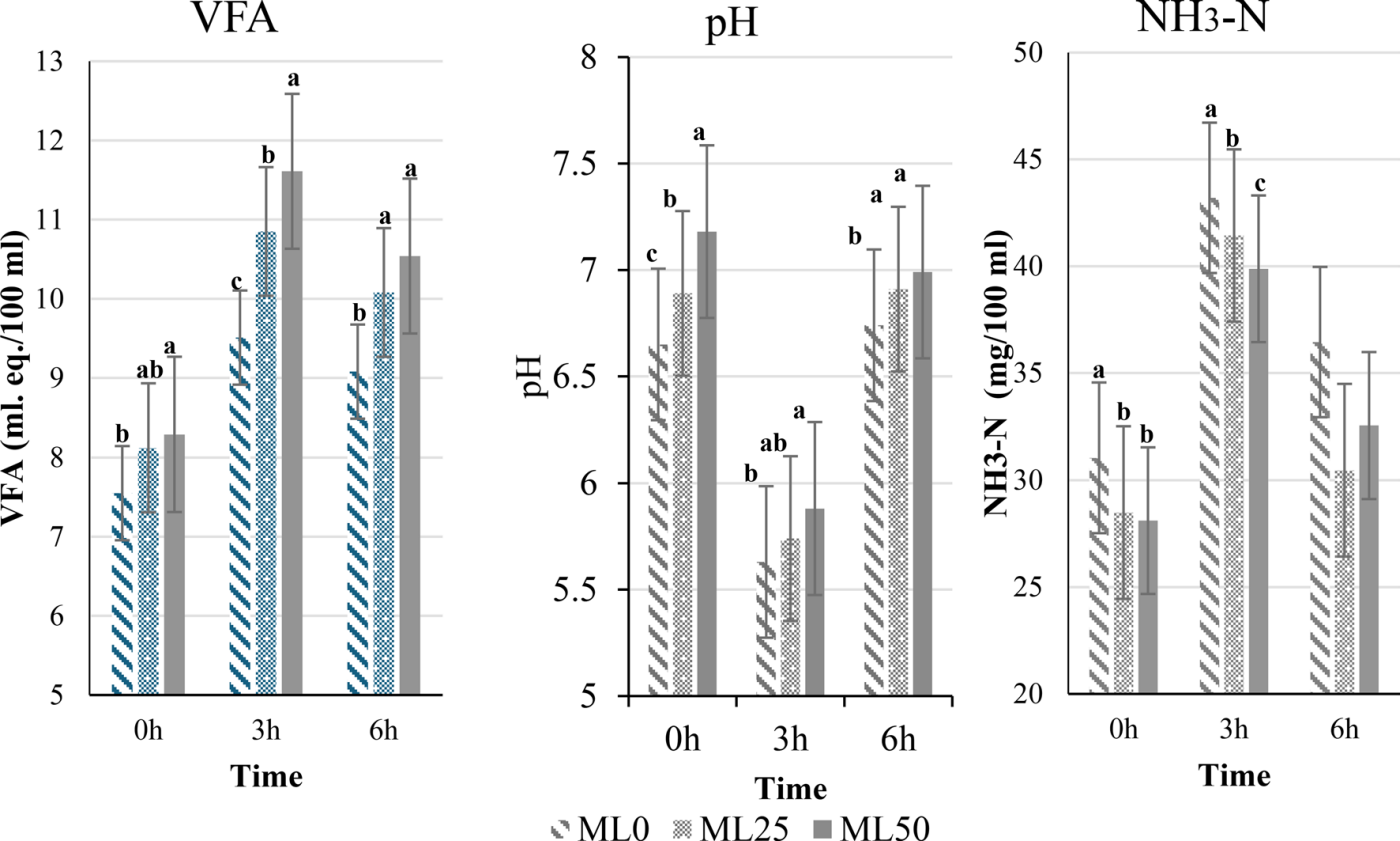
Rumen fermentation activity of goats fed experimental rations. ML0: basal ration (60% concentrate feed mixture (CFM) plus 250 g rice straw/goat/day alongside Berseem (*Trifolium alexandrinum*) as *ad libitum*); ML25; basal ration (ML0) with 25% of the CFM substituted by mulberry leaves; ML50: basal ration (ML0) with 50% of the CFM replaced by mulberry leaves. a, b and c mean that different subscripts differ significantly (*P* < 0.05). VFA: volatile fatty acid (acetate + propionate +butyrate+ valerate +isobutyrate+ isovalerate), NH3-N: ammonia nitrogen

Compared to the control group (ML0), goats consuming mulberry-supplemented diets (ML25 and ML50) showed a significant increase in total volatile fatty acids, especially after 3- and 6-h intake. After ingestion, rumen pH decreased, reaching its lowest point at 3 h in all groups. It subsequently ascended back to normal levels after 6 h. The peak NH₃-N concentrations occurred immediately post-feeding, with the control group (ML0) exhibiting the highest levels. Conversely, ML25 and ML50 exhibited reduced (*P* < 0.05) NH₃-N concentrations at 3 and 6 h (Fig. 2).

### Reproductive performance

During the subsequent postpartum breeding cycle, mulberry-supplemented does, particularly those in the ML50 group, showed improved estrous behavior and reproductive parameters. The results showed a 100% estrus rate in ML50, with estrus duration extending to 35 h (*P* < 0.05), while the interval from buck introduction to estrus considerably decreased from 8.33 days to 4.0 days (*P* < 0.01). The present findings indicate that ML25 and ML50 improved postpartum reproductive responses, as reflected by higher mating, pregnancy, fecundity, and prolificacy rates during the subsequent mating season. (Table 3).

**Table 3:** Effect of experimental rations^1^ on ovarian activity and reproduction traits during the breeding season

Item	ML0	ML25	ML50	SEM	*P* value
Ovarian activity					
Total number of does	12	12	12	--	--
Initial body weight, kg	42.16	42.08	42.16	0.486	0.990
No. of does shows estrus, mating	10	10	12	--	--
Does exhibited estrus, %^2^	83.33^b^	83.33^b^	100^a^	4.314	0.010
Duration of estrus, hour	27^b^	33^ab^	35^a^	2.151	0.035
Days of Estrus, day	8.33^a^	4.66^b^	4.00^b^	0.562	0.001
Reproduction traits					
Total No. of does	12	12	12	--	--
No. of does mate	10	10	12	--	--
Mating rate, %^3^	83.33^b^	83.33^b^	100^a^	4.314	0.010
No. of does pregnant	8	9	10	--	--
Conception rate, %^4^	80.00^b^	90.00^a^	83.33^b^	3.534	0.021
Pregnancy rate, %^5^	66.66^b^	75.00^ab^	83.33^a^	2.084	0.022
No. of does kid	8	9	10	--	--
kidding rate, %^6^	100	100	100	4.095	1.000
No. of born kids	8	10	12	--	--
Fecundity rate, %7	66.66^c^	83.33^b^	100^a^	3.963	0.001
Prolificacy rate, %^8^	100^b^	111.1^ab^	120.00^a^	5.113	0.037
No. of twins	-	1	2	--	--
Twinning rate, %^9^	-	11.11b	20^a^	0.791	0.001

^1^ML0: basal ration (60% concentrate feed mixture (CFM) plus 250 g rice straw/goat/day with Berseem (*Trifolium alexandrinum*) as *ad libitum*); ML25; basal ration (ML0) with 25% of the CFM substituted by mulberry leaves (ML); ML50: basal ration (ML0) with 50% of the CFM replaced by ML

^2^Does exhibited estrus (%) =Total number of does showing estrus (matting) / Total Number of does %

^3^Mating rate (%) = No. of does mated / Total No. of does. ^4^Conception rate (%) = No. of does pregnant / No. of does mate

^5^Pregnancy rate (%) = No. of does pregnant /Total no. of does. ^6^kidding rate (%) = No. of does kidded / No. of does pregnant

^7^Fecundity rate (%) = No. of kids born /Total No. of does. ^8^Prolificacy rate (%) = No. of kids born / No. of does kids

^9^Twining rate (%) = No. of twins / No. of kids

a, b and c mean values in the same row with different subscripts differed significantly (*P* < o.o5). ML0: control diet

**Fig. 3:**
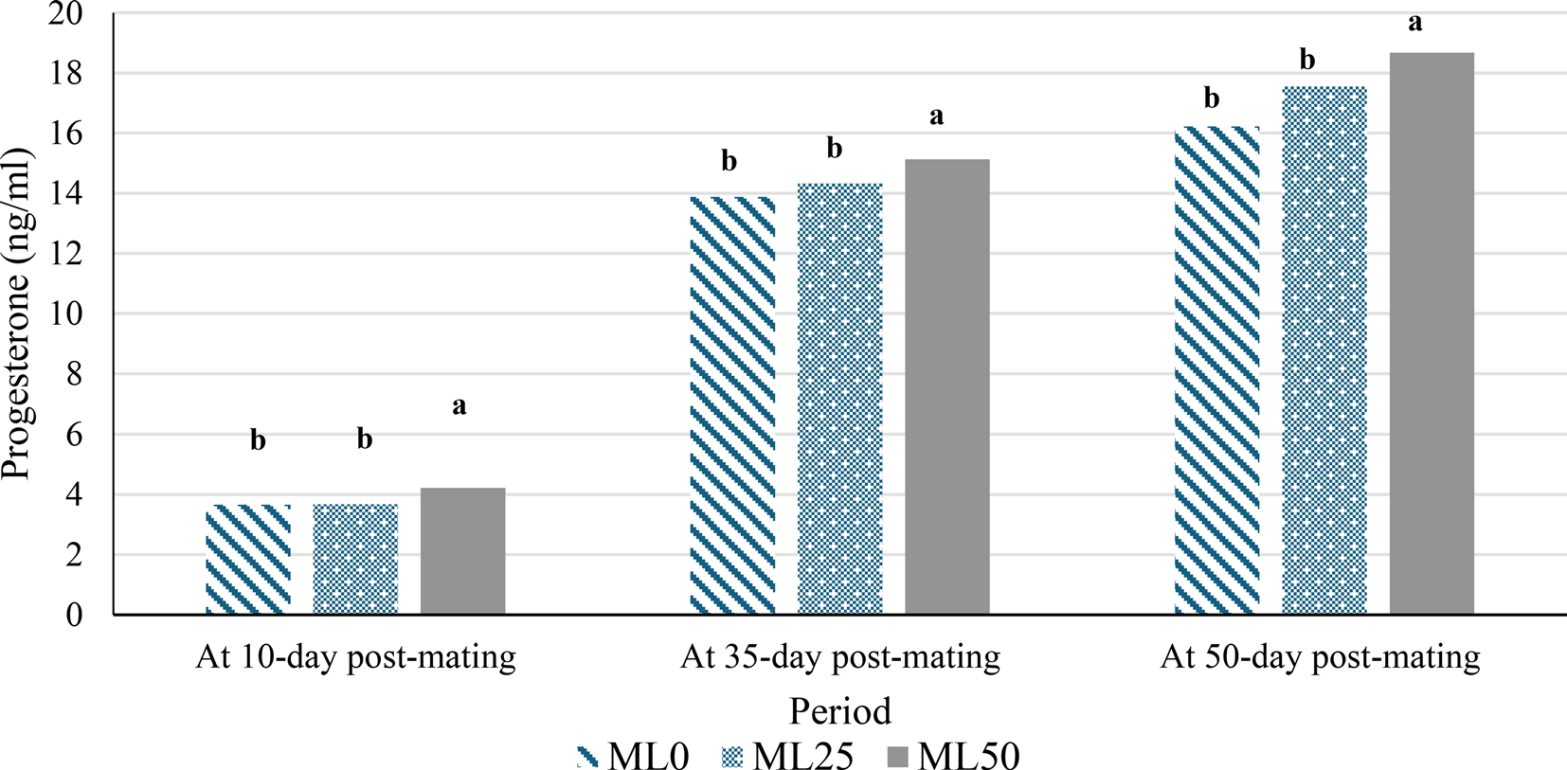
Effect of treatment on progesterone concentration in serum of does at different days post-mating during the breeding season. ML0: basal ration (60% concentrate feed mixture (CFM) plus 250 g rice straw/goat/day alongside Berseem (*Trifolium alexandrinum*) as *ad libitum*); ML25; basal ration (ML0) with 25% of the CFM substituted by mulberry leaves; ML50: basal ration (ML0) with 50% of the CFM replaced by mulberry leaves. a and b: means denoted within the same row with different superscripts are significantly different at *P* < 0.05

The progesterone mechanism (Fig. 3) provides endocrine evidence of improved luteal function in mulberry-fed does: Following copulation, P4 concentrations increased progressively, and at 35 and 50 days, they were significantly elevated in ML50 (*P* < 0.05).

### Growth performance

Data present in Table 4 demonstrated that incorporating ML powder into the ration significantly influenced the growth of does and their offspring. The body weights of does during late pregnancy, parturition, and three months postpartum were comparable among groups. However, the ML50 group had considerably increased (*P* < 0.05) body weights during the first and second months postpartum compared to the control group (ML0). Moreover, mulberry supplementation significantly increased weaning weight in kids, whereas total weight gain and average daily gain showed numerical improvements but did not reach statistical significance. In contrast, litter performance, including litter weight at weaning, total litter weight gain, and litter average daily gain, was significantly improved in the mulberry-fed groups, particularly ML50, compared with the control group.

**Table 4:** Effect of experimental rations^1^ on live body weight and change in body weight of does and their offspring performance during the experimental period

Item	ML0	ML25	ML50	SEM	*P* value
Does performance					
No. of does pregnant	8	9	10	--	--
body weight at Late pregnancy, kg	46.16	46.16	46.33	0.530	0.967
Body weight at parturition, kg	39.33	40.50	40.75	0.417	0.050
Body weight at 1st month after parturition, kg	36.75^b^	38.50^a^	39.25^a^	0.444	0.001
Body weight at 2nd month after parturition, kg	39.41^b^	40.50^b^	41.33^a^	0.409	0.003
Body weight at 3rd month after parturition, kg	42.08	42.50	43.41	0.396	0.050
Changes in body weight after parturition, kg					
Parturition to1st month	-2.58	-2.00	-1.50	0.604	0.458
1st month to 2nd month	2.66^a^	2^b^	2.08^ab^	0.517	0.048
2nd month to 3rd month	2.66	3	2.08	0.440	0.345
parturition to weaning	2.75	2	2.66	0.512	0.558
Offspring performance					
Total number of born kids	8	10	12	--	--
Litter size/ doe at birth (LSB)	1.00	1.11	1.20	--	--
Birth weight, kg	3.75	3.55	3.44	0.262	0.662
Weaning weight kg	15.83	16.23	16.91	1.181	0.048
Total weight gain, (kg	12.08	12.68	13.47	1.267	0.136
Average daily gain, g/ day	134.2	140.9	149.6	0.014	0.136
Relative improvement, %	100	105	111.5	--	--
Does production					
Litter weight at birth, kg	3.75	3.94	4.13	0.185	0.44
Litter weight at weaning, kg	15.83^c^	18.01^b^	20.29^a^	0.447	0.001
Total litter weight gain, kg	12.08^c^	14.07^b^	16.16^a^	0.494	0.001
Average daily gain, g/day	134.2^c^	156.3^b^	179.5^a^	0.005	0.001
Relative improvement, %	|100	116.4	133.7	--	--

^1^ML0: basal ration (60% concentrate feed mixture (CFM) plus 250 g rice straw/goat/day with Berseem (*Trifolium alexandrinum*) as *ad libitum*); ML25; basal ration (ML0) with 25% of the CFM substituted by mulberry leaves (ML); ML50: basal ration (ML0) with 50% of the CFM replaced by ML

a, b and c mean values in the same row with different subscripts differed significantly (*P* < o.o5)

### Milk production and quality

Dietary inclusion of mulberry leaf powder led to a numerical increase in total milk yield and 4% fat-corrected milk (FCM); however, these elevations did not reach statistical significance (Table 5). In contrast, milk quality was significantly influenced by the treatment. Although milk fat and protein percentages were lower in the mulberry-supplemented groups compared with the control group, fat yield was enhanced, while total milk yield and 4% fat-corrected milk yield exhibited only numerical, non-significant increases. While lactose and total solids remained stable across groups, the absolute yields of milk components increased in tandem with the overall production trends, suggesting that mulberry supplementation enhances the partitioning of nutrients toward milk solid synthesis rather than just fluid volume.

**Table 5:** Effect of different experimental rations^1^ on milk yield and its composition

Item	ML0	ML25	ML50	SEM	*P* value
Daily milk yield, g/ h/d	1505	1577	1614	64.95	0.525
Daily 4%-FCM, g/ h/d^2^	1408	1466	1493	75.22	0.694
Milk composition, %					
Fat	3.57^a^	3.53^b^	3.50^b^	0.164	0.001
Protein	4.59^a^	3.95^b^	3.83^b^	0.110	0.001
Lactose	4.11	4.05	3.9	0.072	0.716
Total solids	13.16	12.39	12.04	0.353	0.667
Solids not fat	9.59	8.86	8.55	0.243	0.612
Ash	0.89	0.86	0.81	0.036	0.639
Yield of composition milk, g/h/d					
Fat	53.72^b^	55.66^b^	57.47^a^	3.608	0.011
Protein	69.07^b^	62.29^a^	61.81^a^	3.258	0.033
Lactose	61.85	63.86	62.94	2.748	0.868
Total solids	198.03	195.37	195.29	9.744	0.804
Solids not fat	144.31	139.71	137.82	6.547	0.788
Ash	13.39	13.56	13.07	0.939	0.966

^1^ML0: basal ration (60% concentrate feed mixture (CFM) plus 250 g rice straw/goat/day with Berseem (*Trifolium alexandrinum*) as *ad libitum*); ML25; basal ration (ML0) with 25% of the CFM substituted by mulberry leaves (ML); ML50: basal ration (ML0) with 50% of the CFM replaced by ML

^2^Fat-corrected milk (FCM) for goats was calculated according to Mavrogenis and Papachristoforou (1988) equation, FCM for goat = milk yield (0.411 + 0.147*% fat)

a and b mean values in the same row with different subscripts differed significantly (*P* < o.o5)

### Blood biochemical parameters

**Fig. 4:**
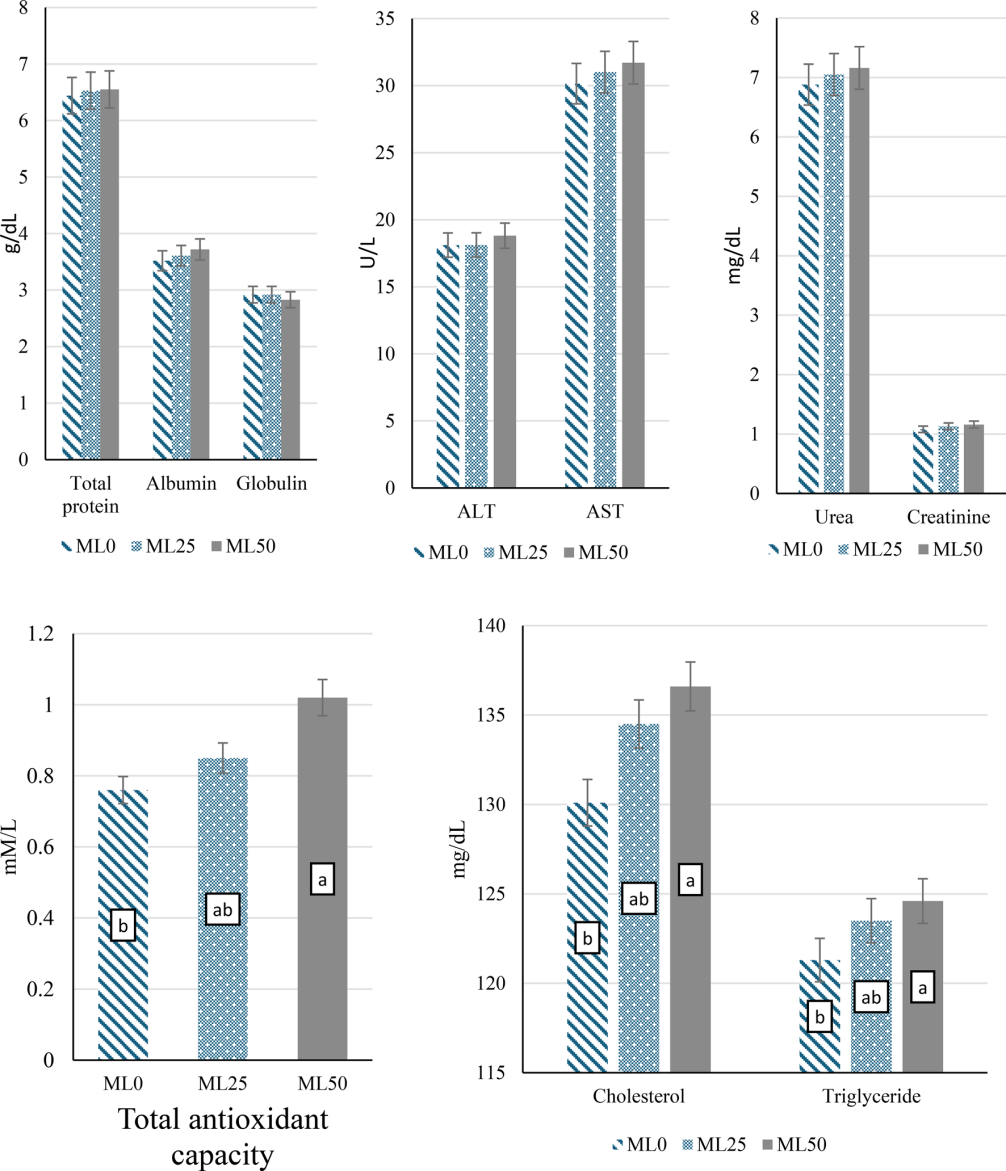
Biochemical parameters in blood plasma as affected by experimental rations^1^. ^1^ML0: basal ration (60% concentrate feed mixture (CFM) plus 250 g rice straw/goat/day with Berseem (*Trifolium alexandrinum*) as *ad libitum*); ML25; basal ration (ML0) with 25% of the CFM substituted by ML. ALT, alanina aminotransferasa; AST, aspartato aminotransferase. a, and b mean values in the same row with different subscripts differed significantly *P* < o.o5

Cholesterol and triglycerides were associated with the ML0 group (*P* < 0.05). In contrast, the other blood components showed no significant differences across the experimental groups. Significant variations (*P* < 0.05) in blood urea nitrogen (BUN) were seen among the groups. The concentration of BUN, as the final product of protein catabolism and proteolysis, is directly influenced by the quantity of CP in the diet. BUN correlates negatively with the body’s ability to store nitrogen and consume protein. The observed results (*P* < 0.05) suggest that the inclusion of ML in the ration enhances nutritional consumption, resulting in increased blood protein and urea levels (Fig. 4).

### Feed efficiency

**Fig. 5:**
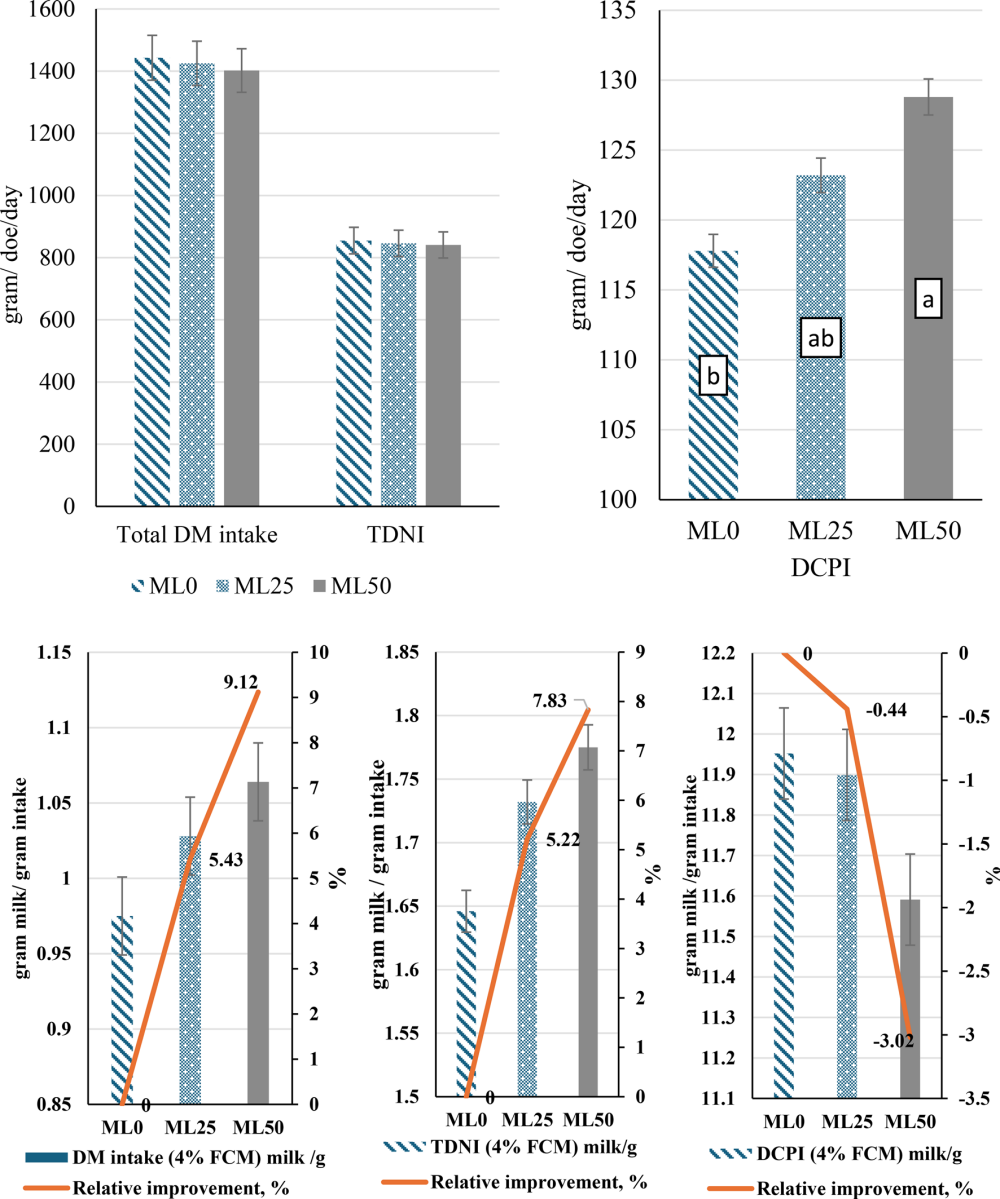
Feed efficiency of milk production during the suckling period (90 days). ML0: basal ration (60% concentrate feed mixture (CFM) plus 250 g rice straw/goat/day with Berseem (*Trifolium alexandrinum*) as *ad libitum*); ML25; basal ration (ML0) with 25% of the CFM substituted by ML. DM: Dry matter; TDNI: total digestible nutrients intake; DCPI: digestible crude protein intake; FCM 4%/; Fat corrected milk 4%. a and b mean values in the same row with different subscripts differed significantly *P* < o.o5

Incorporating mulberry leaves into the diet significantly enhanced efficiency (g FCM4%/g DM), with groups ML25 and ML50 showing increases of 5.4% and 9.1%, respectively, compared with the control group. Feed efficiency, quantified as grams of FCM4% per gram of total digestible nutrients (g FCM4%/g TDN), increased by 5.2% and 7.8% in groups ML25 and ML50, respectively. When calculated per unit of digestible crude protein (g FCM4%/g DCP), feed efficiency decreased slightly in ML25 (− 0.44%) and more significantly in ML50 (− 3.02%) relative to the ML0 group. The overall enhancement in feed efficiency was influenced (*P* < 0.05) by the increased dietary inclusion of mulberry leaves (Fig. 5).

### Partial farm budget (economic evaluation)

The results, of the partial farm budget analysis for incorporating mulberry leaves, clearly indicate that incorporating increased mulberry leaves into the diet reduced the total feed costs relative to the control group. The 50% mulberry leaf inclusion (ML50) achieved the highest return on investment (ROI), yielding 4.3 LE per unit of feed cost. The 25% inclusion level (ML25) ranked second, with an ROI 2.86 LE per unit of feed cost. Both treatments outperformed the control group (ML0), which recorded a ROI 2.1 LE per unit of feed expenditure (Table 6).

**Table 6:** Economic evaluation^1^ of dams during the suckling period (90 days)

Item	ML0^2^	ML25^2^	ML50^2^
Total number of dams	8	9	10
Average daily feed intake, kg/dam/day (as fed)			
CFM	1.000	0.750	0.500
Mulberry leaves	---	0.250	0.500
Rice straw	0.250	0.250	0.250
Barseem	1.822	1.755	1.677
Total feed intake, kg/dam/90 days (as fed)			
CFM	90	67.5	45
Mulberry leaves	---	22.5	45
Rice straw	22.5	22.5	22.5
Barseem	163.98	157.95	150.93
Average total feed cost, LE/dam/90 days			
CFM	1260	945	630
Mulberry leaves	---	33.75	67.50
Rice straw	9	9	9
Barseem	147.58	142.15	135.83
Total feed cost, LE/dam/90 days— input	1416.58	1129.90	842.33
Total feed cost, LE/kids/90 days— input	1416.58	1017.92	701.94
Dam production at weaning			
Litter size/doe	1.00	1.11	1.20
Litter weight at weaning, kg	15.83^b^	18.01^ab^	20.29^a^
Price of kid born (180 LE/ kg) /dam— output	2849.4	3241.8	3652.2
Price of kid born (180 LE/ kg) --- output	2849.4	2920.5	3043.5
Net revenue (LE/dam/90 days) A	1432.8	2111.9	2809.8
Net revenue (LE/kids born/90 days) A	1432.8	1902.6	2341.5
Economic efficiency/dam B	2.01	2.86	4.33
Economic efficiency/kids born B	2.01	2.86	4.33
Relative improvement (%)	100	142.6	215.4

^1^Prices of concentrate feed mixture (CFM), Berseem, Rice straw, and Mulberry leaves were 14,000, 900,400 and 1500 L.E./ton, respectively. Price of kg live body weight per 180 LE based on the market price in 2024.Total daily feed cost (L.E.) = (cost of CFM+ Rice straw + Berseem + Mulberry leaves). A Net revenue (LE/ewe/day) = money output – money input. B Economical efficiency = money output/money input

^2^ML0: basal ration (60% concentrate feed mixture (CFM) plus 250 g rice straw/goat/day with Berseem (*Trifolium alexandrinum*) as *ad libitum*); ML25; basal ration (ML0) with 25% of the CFM substituted by mulberry leaves (ML); ML50: basal ration (ML0) with 50% of the CFM replaced by ML

a, b and c mean values in the same row with different subscripts differed significantly (*P* < o.o5)

## Discussion

The active compound in Fig. 1 shows that the substantial presence of chlorogenic acid is crucial, as it serves as a potent antioxidant and helps regulate glucose and lipid metabolism, thereby reducing oxidative stress and metabolic irregularities. Gallic acid and catechin, in abundance, are recognized for their potent antioxidant, antibacterial, and anti-inflammatory properties, which may help maintain animal health and microbial equilibrium. Syringic acid, a significant phenolic compound, is associated with hepatic protection and the regulation of metabolic pathways. Even minor components may work together to enhance bioactivity. Flavonoids such as rutin, quercetin, and kaempferol help protect blood vessels, regulate enzymes, and enhance nutritional absorption. Conversely, ellagic and rosmarinic acids exhibit superior antioxidant and antibacterial properties. Both caffeic and ferulic acids are chemicals linked with anti-inflammatory properties. These acids are less prevalent than other chemicals; nevertheless, they improve the overall bio-functional quality of ML. The high phenolic content underscores the potential of Morus alba leaves as a significant source of natural antioxidants and bioactive compounds, with important implications for improving metabolic efficiency, enhancing health, and reducing oxidative stress in animals.

Incorporating mulberry leaves (ML) into the diet significantly enhanced the digestion of OM, CP, and CF. neutral detergent fiber (NDF) and acid detergent fiber (ADF) exhibited significant enhancements, indicating that the impact on nutrient utilization was dose-dependent. The digestibility of dry matter (DM), ether extract (EE), and nitrogen-free extract (NFE) remained unchanged; however, the digestibility of total digestible nutrients (TDN), digestible crude protein (DCP), and DCPI varied, indicating that these advantages resulted from enhanced digestibility rather than increased feed intake. The observed effects are likely linked to the phytonutrients and high phenolic content of Morus alba leaves, encompassing flavonoids and phenolic acids, which act as natural antioxidants, augment digestive enzyme activity, and enhance metabolic efficiency (Kong et al. 2019; Ma et al. 2017). Other ruminants have similarly experienced advantages from ML supplementation, enhancing the digestibility of DM, OM, total nitrogen, NDF, and ADF, in addition to promoting rumen epithelial growth and ruminal fermentation (Ouyang et al. 2019; Huyen et al. 2012; Doran et al. 2007). Furthermore, the incorporation of ML has been associated with improved feed consumption, immune function, growth performance, and mammary gland development, highlighting its potential as a functional feed additive (Geng et al. 2024).

The ML50 goats, with the highest inclusion level, had the highest VFA levels. This suggests that microbial activity was raised, and glucose fermentation followed more rapidly. The increased availability of VFA provides additional energy precursors for milk synthesis and development, which correlates with the advanced feed efficiency observed in ML25 and ML50. Ouyang et al. (2019) found similar results, which involved mulberry in the feeding system, led to improved fermentation effectiveness and nutrient utilization. The post-ingestion pH trend was similar across all groups; however, goats in ML25 and ML50 groups demonstrated significantly advanced pH levels than those in ML0 group, remaining within the optimal range (6.2–6.8). The buffering effect is linked to mulberry’s secondary metabolites, mainly tannins and flavonoids, which adjust microbial fermentation and avoid excessive acid gathering (Li et al. 2022). Also, sustaining rumen stability by reduces the likelihood of subacute ruminal acidosis and facilitates rapid degradation of fiber and starch (Hassan et al. 2020). The peak NH₃-N concentrations occurred immediately post-feeding, with the control group (ML0) exhibiting the highest levels; conversely, ML25 and ML50 revealed significantly lower NH₃-N concentrations at 3 and 6 h (*P* < 0.05). These results indicate their superior capacity for ammonia adjustment throughout microbial protein synthesis. Thus, this increase is possible attributable to amended synchronization between the release of energy from carbohydrate fermentation and the supply of nitrogen. Consequently, the body utilizes protein more effectively and excretes less nitrogen in urine. These results align with Li et al. (2020), who demonstrated that the incorporation of mulberry enhanced nitrogen metabolism. The beneficial effects of including mulberry in the diet mostly stem from its impact on the microbial composition of the rumen. Rumen microorganisms ferment feed to generate VFA (Stewart et al. 1997). Studies demonstrate that mulberry leaves increase the abundance of cellulolytic bacteria (Tan et al. 2012) and total bacterial populations (Niu et al. 2016), thereby promoting enhanced fiber decomposition. Huyen et al. (2012) stated that mulberry enhances the ruminal microenvironment, confirming our findings. Elevated concentrations of VFA promote the growth of rumen papillae, hence enhancing the rumen’s capacity for nutrient absorption over time (Gäbel et al. 1999). The phenolic constituents of *Morus alba* (Fig. 1) clarify the molecular foundation for these findings. Elevated concentrations of chlorogenic acid and catechin enhance cellulolytic and amylolytic bacterial activity, contributing to the increased VFA levels in ML25 and ML50 (Tan et al. 2012; Niu et al. 2016). Additional phenolics, including gallic acid, syringic acid, and rutin, regulate microbial populations by inhibiting lactate producers and facilitating lactate consumers, so maintaining pH within physiological parameters. The reduction of NH₃-N in mulberry-fed groups (Fig. 2) may be ascribed to chlorogenic acid, gallic acid, and quercetin, which enhance microbial nitrogen assimilation by inhibiting proteolysis and deamination, while promoting nitrogen incorporation into microbial protein (Huyen et al. 2012; Li et al. 2020).

Furthermore, flavonoids such as quercetin, kaempferol, and rutin provide antioxidant protection for ruminal bacteria despite their lower concentrations. This enhances the efficacy of fermentation. The phenolic-rich composition of mulberry leaves (Fig. 1) resulted in enhanced rumen fermentation patterns (Fig. 2), evidenced by increased volatile fatty acids (enhanced energy availability), more stable ruminal pH (safer environment for fermentation), and reduced ammonia nitrogen (improved nitrogen use). These combinatorial advantages result in improved milk composition, enhanced feed efficiency, and increased earnings, while simultaneously reducing nitrogen excretion. Mulberry leaves constitute a valuable plant-based supplement for sustainable ruminant production.

In line with earlier research showing that dietary and phytochemical supplementation improve ovarian function and reproductive performance, Table 3 shows notable improvements in postpartum estrus behavior and reproductive parameters during the subsequent mating season in mulberry-supplemented does (ML25 and particularly ML50). This study showed a 100% estrus rate in ML50, with estrus duration extending to 35 h (*P* < 0.05), while the interval from buck introduction to estrus considerably decreased from 8.33 days to 4.0 days (*P* = 0.001). These alterations align with Bisinotto et al. (2012) and Abuelo et al. (2019), who demonstrated that improved energy availability and antioxidant protection promote cyclicity and shorten the interval to estrus. Li et al. (2020) found that administering mulberry flavonoids to ruminants increased reproductive hormone levels and fertility. This corroborates our findings that ML25 and ML50 exhibited higher mating, pregnancy, fecundity, and prolificacy rates.

Improved luteal function in does fed mulberries is demonstrated endocrinologically via the progesterone pathway (Fig. 3). The concentrations of P4 gradually increased after copulation, reaching a considerably higher ML50 at 35 and 50 days (*P* < 0.05). This pattern, characterized by elevated, sustained P4, has previously been linked to the antioxidant and luteo-protective properties of dietary bioactive compounds (Rebai et al. 2017). Thus, present data confirm that mulberry phytochemicals improve corpus luteum function and facilitate pregnancy retention, consistent with studies showing increased blood P4 levels and improved pregnancy outcomes following phytogenic consumption. The phenolic profile of mulberry leaves offers a plausible molecular rationale for these reproductive responses. Elevated concentrations of chlorogenic acid and catechin, in conjunction with gallic and syringic acids and minor flavonoids (quercetin, rutin, kaempferol), are acknowledged to augment rumen fermentation and energy accessibility, alleviate oxidative stress in reproductive tissues, and modulate endocrine signals pertinent to follicular development and luteal preservation (Tan et al. 2012; Niu et al. 2016; Li et al. 2020). The combined effects account for ML50’s superior estrus expression, elevated P4 secretion, and enhanced fertility markers compared to the control group. Similar mechanistic relationships among enhanced nutritional status, reduced oxidative damage, and increased reproductive function have been observed in ruminants fed high-energy or antioxidant-rich diets (Zabuli et al. 2010; Taye et al. 2013). The present findings extend previous observations by illustrating that a substantial inclusion level (50% mulberry leaf) can effectively enhance many reproductive parameters (mating, conception, pregnancy, prolificacy, and twinning) without significant adverse effects. These results are supported by the findings of Li et al. (2020) and Kumar et al. (2015), which demonstrated improved reproductive hormones and offspring outcomes after mulberry consumption, as well as by Rebai et al. (2017), which reported antioxidant-mediated luteal support. This study supports the use of mulberry leaves as an efficient phytogenic method to improve reproductive function in goats through combined nutritional and biochemical processes.

Table 4 showed that adding ML powder to the feed significantly affected doe and offspring growth. The groups had identical body weights during late pregnancy, parturition, and three months postpartum. Compared to the control group (ML0), the ML50 group showed a substantial rise (*P* < 0.05) in body weights during the first and second months postpartum. Weaning weight, total increase, and ADG improved with supplements in kids. The mulberry-fed groups (ML25 and ML50) showed significant improvements (*P* < 0.05) in litter performance, including weaning weight, growth, and ADG, compared to the control group. These findings align with those of Luo et al. (2025), who demonstrated that mulberry leaves improve growth performance by increasing nutrient digestibility, facilitating rumen fermentation, and elevating antioxidant capacity. The phenolic composition of mulberry leaves (Fig. 1) explains the mechanisms causing these enhancements. Increased levels of chlorogenic acid and catechin in the rumen accelerate the fermentation of carbohydrates and fiber, thereby increasing the production of VFA, which are essential for tissue development. The extra energy rapidly improves weight gain and accelerates growth. Gallic acid and syringic acid exhibit significant antioxidant effects, protecting tissues from oxidative stress and enhancing metabolic efficiency during the increased energy demands of late pregnancy and early lactation. Flavonoids like quercetin, rutin, and kaempferol support hormone regulation and enhance nutrient absorption, thereby facilitating growth in kids and improving litter performance. The bioactive compounds illustrated in Fig. 1clarify the mechanistic foundation for the observed enhancement of growth traits in the present study by enhancing energy metabolism through improved rumen fermentation (chlorogenic acid, catechin), reducing oxidative stress and promoting tissue growth (gallic and syringic acids), and affecting nutrient utilization and hormonal regulation (quercetin, rutin, kaempferol). The results indicate that ML serves as a sustainable feed supplement that promotes the mother’s weight recovery and enhances offspring growth due to its high phenolic compound content.

The incorporation of mulberry leaf powder into goat feed led to a numerical increase in both total milk yield and 4% fat-corrected milk (FCM); however, these changes did not reach statistical significance (Table 5). While the total volume of milk remained relatively stable, its chemical composition exhibited significant improvements, particularly in protein and fat contents compared to the control (ML0) group. These findings suggest that mulberry supplementation optimizes nutrient utilization efficiency specifically for the synthesis of milk solids, rather than mere fluid volume, which aligns with Li et al. (2020), who observed enhanced milk production efficiency in buffaloes. Notably, although the relative percentages of protein and fat showed variation, their absolute yields increased in parallel with total production trends, emphasizing the importance of evaluating both concentration and absolute yield in dairy research. Consistent with Kumar et al. (2015), who reported substantial increases in milk protein and fat in goats supplemented with mulberry, the improvement observed in this study is directly attributable to the bioactive substances identified in ML (Fig. 1). Specifically, chlorogenic acid and catechin play a pivotal role by enhancing nutrient digestibility and accelerating ruminal fermentation, thereby increasing the availability of energy precursors for milk synthesis. Simultaneously, gallic and syringic acids act as potent antioxidants that mitigate oxidative stress during lactation, maintaining metabolic homeostasis. Furthermore, flavonoids such as quercetin, rutin, and kaempferol are essential for supporting mammary gland function through hormonal modulation and cellular protection. Collectively, the phenolic and flavonoid profile identified in this study provides a robust mechanistic rationale for the improved yields, confirming that mulberry leaves serve as an effective natural supplement for enriching milk quality and promoting sustainable dairy production (Li et al. 2020; Kumar et al. 2015).

Figure 4 shows blood biochemistry. Consuming 50% mulberry leaves resulted in higher levels of TAC, cholesterol, and triglycerides compared to the ML0 group (*P* < 0.05). Other blood components did not differ between experimental groups. These haematological parameters reflect the biochemical alterations that occur during the consumption of various feeds, primarily due to the functioning of metabolic pathways and their interaction with ruminal fermentation. Changes in specific blood parameters can indicate that dietary supplements may have a beneficial effect. Significant variations (*P* < 0.05) in BUN were seen among the groups. The concentration of BUN, as the final product of protein catabolism and proteolysis, is directly influenced by the quantity of CP in the diet. BUN correlates negatively with the body’s ability to store nitrogen and consume protein. The observed results (*P* < 0.05) suggest that the inclusion of ML in the ration enhances nutritional consumption, resulting in increased blood protein and urea levels. Ma et al. (2017) noted analogous findings, associating improved digestion of OM and nitrogen with increased levels of blood total protein and urea. Mulberry leaves are acknowledged for their substantial nutrient composition and varied assortment of bioactive phytochemicals, including anthocyanins, stilbenes, flavonoids, terpenoids, and jasmonic acid (Liu et al. 2010; Casano et al. 2010). These compounds exhibit a diverse array of biological activities, encompassing antibacterial, antipyretic, anticancer, antioxidant, hypoglycemic, and metabolism-enhancing properties (Zhou et al. 2014; Kumkoon et al. 2023).

Feed efficiency (g FCM4%/g TDN) increased 5.2% and 7.8% in groups ML25 and ML50, respectively (Fig. 5). Compared to the ML0 group, feed efficiency fell marginally in ML25 (-0.44%) and significantly in ML50 (-3.02%) per unit of digestible crude protein (g FCM4%/g DCP). The overall enhancement in feed efficiency was significantly influenced by the increased dietary inclusion of mulberry leaves (*P* < 0.05). These findings agree with those of Ouyang et al. (2019), who demonstrated that incorporating mulberry leaves at concentrations up to 60% significantly improved feed efficiency. The positive effect may be due to the ability of mulberry leaves to enhance microbial activity in the rumen, thus enhancing nutrient digestibility and ultimately encouraging growth and productivity.

The results in Table 6 indicate that mulberry leaves provide significant potential to improve the cost-efficiency of goat production systems. The economic evaluation indicated that including mulberry leaves in up to 50% of the diet reduced feeding costs by 20% to 50% and enhanced ROI by 40% to 112%. This enhancement can be attributed to two factors: reduced dependence on conventional, costly feed components and the nutritional advantages of ML, which promote growth and enhance the nutritional value of the feed. Including ML in the feed reduces production costs and enhances overall profitability. This strategy is significant as it promotes the utilization of locally sourced, cost-effective, and nutritionally rich feed resources. This enhances the sustainability of animal output and increases farm resilience to fluctuations in feed prices.

## Conclusion

The study concludes that substituting 50% of the concentrate mixture with *Morus alba* (mulberry) leaves may represents a sustainable and economically viable strategy for improving the productivity of Damascus goats. The bioactive phenolic of mulberry leaves, particulary chlorogenic acid and catechin, may contribute to improved ruminal fermentation, as reflected by increased total volatile fatty acid production. These responses were associated with improved fat yield, better offspring growth performance, and enhanced postpartum reproductive responses included higher progesterone levels. Moreover, mulberry leaves mitigate postpartum oxidative stress, supporting maternal body condition recovery and maximizing overall economic returns for livestock producers.

## Data Availability

Not applicable.

## Declarations

### AI assistance declaration

The authors exploited ChatGPT for language editing and assume full responsibility for the final contents.
